# Complete Chloroplast and Mitochondrial Genome Sequences of the Hydrocarbon Oil-Producing Green Microalga *Botryococcus braunii* Race B (Showa)

**DOI:** 10.1128/genomeA.00524-16

**Published:** 2016-06-09

**Authors:** Olga Blifernez-Klassen, Daniel Wibberg, Anika Winkler, Jochen Blom, Alexander Goesmann, Jörn Kalinowski, Olaf Kruse

**Affiliations:** a Center for Biotechnology (CeBiTec), Bielefeld University, Bielefeld, Germany; b Bioinformatics and Systems Biology, Justus-Liebig-University Gießen, Gießen, Germany

## Abstract

The green alga *Botryococcus braunii* is capable of the production and excretion of high quantities of long-chain hydrocarbons and exopolysaccharides. In this study, we present the complete plastid and mitochondrial genomes of the hydrocarbon-producing microalga *Botryococcus braunii* race B (Showa), with a total length of 156,498 and 129,356 bp, respectively.

## GENOME ANNOUNCEMENT

Today, microalgae are generally considered a sustainable, carbon-neutral biofuel feedstock that represents an alternative ecofriendly resource compared to fossil fuels (1, 2). Among other oleaginous eukaryotic algae, *Botryococcus braunii*, belonging to the class of *Treboxyophyceae*, is capable of the production and excretion of high quantities of polysaccharides and long-chain hydrocarbons (3), whose size and type depends on the distinct race of this alga (4). Many efforts have been made so far to further the understanding of how and which cellular biochemical processes are underlying hydrocarbon and polysaccharide biosynthesis, therefore the acquisition of organelle genomes provides useful information for further investigations.

For the establishment of the *Botryococcus braunii* race B (Showa) organelle genome sequences, purified DNA was used to construct a paired-end sequencing library (Illumina, USA). The obtained sequence reads (2 × 250 bp) were assembled using the GS De Novo Assembler software (version 2.8, Roche, Mannheim, Germany), which resulted in one contig for each replicon. Subsequently, for the circularization an *in silico* gap closure approach (5–7) was applied, resulting in circular replicons for both plastid and mitochondrial genome sequences of *Botryococcus braunii* Showa. Annotation of the replicons was performed within the GenDB2.0 system including a manual refinement (8).

The chloroplast genome (cpDNA) has a length of 156,498 bp and a G+C content of 41.51%. It contains 105 putative protein-coding regions, 31 tRNA, and 3 rRNA genes. We were able to functionally annotate 81 protein-coding genes including 32 photosynthesis-related genes such as photosystem I and II proteins. In comparison with the published 172.83-kb chloroplast genome of *B. braunii* strain SAG 807-1 (9), the Showa cpDNA is slightly smaller, and shares the same order and set of conserved genes except for two (*petL* and orf226). Based on the average nucleotide identity (ANI) of 96.5 % (10, 11), both plastid genomes are closely related.

The mitochondrial genome (mtDNA) of *B. braunii* Showa has a length of 129,356 bp and a G+C content of 50.41%. To date, it is the largest sequenced mitochondrial genome among the *Chlorophyta* phylum, since it contains large open reading frames (ORFs) for proteins with unknown functions, and, additionally seems to be very rich in noncoding regions. It comprises 23 tRNAs, 3 rRNAs, and 43 putative protein coding genes, including 18 ATP synthase and respiratory chain components as well as *tatC* gene. The gene order and content is similar to the mitochondrial genome of another recently published *Botryococcus braunii* Showa strain (84.58 kb [12]), however, based on ANI analysis both mitochondrial genomes show only 75.9% similarity (10, 11). Similar results could be observed for two *Dunaliella salina* strains (13), thus nicely reflecting the high diversity of mtDNA genomes (14, 15).

Chloroplast and mitochondrial organelles play an essential role in energy metabolism of the cell, hence the genome data of the organelles of *Botryococcus braunii* race B (Showa) would be useful for further genetics studies as well as taxonomic and phylogenetic analysis.

### Nucleotide sequence accession numbers.

The cpDNA and mtDNA genome sequences were deposited in GenBank under accession numbers LT545991 and LT545992, respectively.
